# Predictors of Developing Heart Failure in Adults with Congenital Heart Defects

**DOI:** 10.31083/j.rcm2403085

**Published:** 2023-03-08

**Authors:** Kambiz Norozi, Matthias J. Müller, Chuce Xing, Michael R. Miller, Jonas Bock, Thomas Paul, Siegfried Geyer, Claudia Dellas

**Affiliations:** ^1^Department of Pediatrics, Pediatric Cardiology, Western University, London, ON N6A 3K7, Canada; ^2^Department of Pediatric Cardiology, Intensive Care Medicine and Neonatology, University Medical Center, 37075 Goettingen, Germany; ^3^Department of Pediatric Cardiology and Intensive Care Medicine, Hannover Medical School, 30625 Hannover, Germany; ^4^Children Health Research Institute, London, ON N6A 5W9, Canada; ^5^Medical Sociology Unit, Hannover Medical School, 30625 Hannover, Germany

**Keywords:** adult congenital heart disease, heart failure, echocardiography, exercise testing

## Abstract

**Background:**

The population of adults with congenital heart defects (ACHD) is growing. The leading cause of premature death in these patients is heart failure (HF). However, there is still limited information on the predictive factors for HF in ACHD patients.

**Objectives:**

This study re-examined a group of patients with repaired or palliated congenital heart defects (CHD) that were initially studied in 2003. A follow-up period of 15 years has allowed us to identify and evaluate predictors for the development of HF in ACHD.

**Methods:**

All patients with repaired or palliated CHD who participated in the initial study (n = 364) were invited for a follow-up examination. The effects of maximum oxygen uptake (${\mathrm{VO}}_{\text{2max}}$) during exercise stress testing, the cardiac biomarker N-terminal pro brain natriuretic peptide (NT-proBNP), and QRS complex on the development of HF during the follow-up period were investigated.

**Results:**

From May 2017 to April 2019, 249 of the initial 364 (68%) patients participated in the follow-up study. Of these, 21% were found to have mild CHD, 60% had moderate CHD, and 19% had complex CHD. Significant predictors for the development of HF were: NT-proBNP level >1.7 times the upper normal limit, ${\mathrm{VO}}_{\text{2max}}$ <73% of predicted values, and QRS complex duration >120 ms. Combination of these three parameters resulted in the highest area-under-the-curve of 0.75, with a sensitivity of 75% and specificity of 63% for predicting the development of HF.

**Conclusions:**

In this cohort of ACHD patients, the combination of ${\mathrm{VO}}_{\text{2max\%}}$, NT-proBNP, and QRS duration was predictive of HF development over a 15-year follow-up period. Enhanced surveillance of these parameters in patients with ACHD may be beneficial for the prevention of HF and early intervention.

## 1. Introduction 

The prevalence of congenital heart defect (CHD) in newborns is approximately 1% [1]. Improvements in diagnostics and in surgical and catheter interventions over the last 40 years have significantly prolonged life expectancy. As a consequence, the population of adults with congenital heart defects (ACHD) has surpassed the number of children with CHD [2] and continues to increase. However, ACHD experience residual effects and sequelae that require regular surveillance throughout their lifespan [3], including heart failure (HF) and arrhythmias [4].

HF is the leading cause of premature death in ACHD [5]. The identification of surrogate parameters for HF in ACHD and the development of predictive models should lead to earlier identification of high-risk patients, thereby enabling timely intervention and preventive measures.

Although several studies have described prediction models and risk factors for HF in patients without CHD, there is still very little data on predictors for HF in ACHD [6, 7]. The focus of published literature is primarily on the evaluation of a single clinical parameter for predicting long-term outcomes in ACHD, such as N-terminal pro brain natriuretic peptide (NT-proBNP), cardiopulmonary exercise testing, QRS duration, fractional shortening, and ejection fraction [8, 9, 10, 11]. Gaps remain in our understanding of the predictors for HF in ACHD, including the utility of combining various parameters, and whether the available clinical variables can predict HF at an early stage.

To fill this knowledge gap, the aim of the current study was to determine whether a combination of clinical parameters can identify ACHD patients who are at increased risk of HF, therefore requiring more intensive follow-up care and early preventive interventions.

## 2. Methods 

### 2.1 Study Patients

A previous study from 2003–2004 entitled *Life Chances 1* (LC1) investigated 364 patients with various types of repaired or palliated CHD [12]. These patients were treated by surgical or interventional procedures at the University Hospital of Goettingen, Germany, and then followed up in our ACHD clinic. The LC1 study included patients aged 13–50 years (median age, 26.4 years). Patients had undergone a medical history review, physical examination, electrocardiography (ECG), 2D-echocardiogram, blood sampling, metabolic exercise testing, and assessment of their psychological and socioeconomic status.

The present study is entitled *Life Chances 2* (LC2). In 2017, all patients who had participated in LC1 were invited by phone, mail, or their family physician to attend the ACHD clinic for a follow-up examination [13].

The severity of CHD was assessed according to the 2020 European Society of Cardiology (ESC) Guidelines for the management of ACHD [14]. Patients were categorized into three groups: mild, moderate, or complex CHD. In patients with multiple cardiac lesions, the lesion with the highest complexity was used to assign the patient.

### 2.2 Heart Failure 

HF is not a single pathological diagnosis, but rather a clinical syndrome consisting of cardinal symptoms such as breathlessness, ankle swelling, and fatigue. For the purpose of this study, patients were classified as having developed HF if at least one of the following criteria was fulfilled:

(1) Patient had not been taking any HF medication (e.g., diuretic, beta blocker, ACE inhibitor, etc.) during LC1, but then started taking HF medication between LC1 and LC2. Although in a small number of patients these medications might have been used to treat hypertension, this condition could be considered a precursor for HF.

(2) Patient who required a surgical/interventional procedure for their underlying CHD, or had been admitted to a hospital for HF between LC1 and LC2.

(3) Patient who had died of HF between LC1 and LC2.

### 2.3 Inclusion and Exclusion Criteria 

Only patients who had participated in the LC1 study were eligible for inclusion in LC2. Patients who were pregnant during the enrollment period for LC2 were excluded.

### 2.4 Informed Consent 

All patients provided written informed consent. The first part of this study was reviewed and approved by the ethics committee of Hannover Medical School under no. 3710 (date: 04-10-2004) and by the University Medical Center Goettingen under no. 10/2/01 (date: 01-03-2001). The second part was reviewed and approved by the ethics committee of the University Medical Center Goettingen under no. 15/8/14.

### 2.5 Clinical Assessment

All patients underwent physical examination and measurement of heart rate, blood pressure, body weight, height, and standard 12-lead ECG.

#### 2.5.1 Exercise Testing

Exercise testing for LC2 was performed on an upright bicycle ergometer and began with 2 minutes of unloaded peddling, followed by cycling against increasing resistance until exhaustion (RAMP protocol), and concluding with 3 to 5 minutes of cycling with minimal resistance. The choice of ramp protocol steepness was tailored to the patient’s exercise tolerance based on previous exercise tests, gender and weight. The aim was for a test duration ranging between 8 and 12 minutes. Oxygen uptake was measured using breath-by-breath analysis (Oxycon pro, Jaeger Company, Hoechberg, Germany) throughout the exercise procedure. All patients exercised to maximum exercise capability, and peak oxygen consumption (${\mathrm{VO}}_{\text{2max}}$) was determined as the highest value in the terminal phase of exercise. In the present study, the percentage of predicted ${\mathrm{VO}}_{\text{2max}}$ rather than its absolute value was used in order to eliminate the impacts of age, gender, and body mass index. A 12-lead ECG was recorded continuously during exercise testing. Blood pressure was recorded every 2 minutes using an automated cuff sphygmomanometer. In LC1, a conventional (STEP) protocol was applied with a 25 Watt increase in the work-load every 2 minutes until exhaustion, as described previously. Michalik *et al*. [15] found that both protocols were comparable in terms of achieving maximal fat oxidation and maximal heart rate. However, the peak power output reached in the STEP test was significantly lower, albeit slightly, compared to the RAMP protocol (388.0 ± 39.9 W vs. 406.1 ± 44.8 W, respectively; *p *< 0.05) [16].

#### 2.5.2 NT-proBNP Measurement 

Peripheral venous blood samples were obtained from all patients after resting for at least 15 minutes and prior to exercise testing. The blood samples were immediately placed on ice and centrifuged at 5000 rpm for 10 minutes. Plasma and serum aliquots were stored at –80 °C until further analysis. NT-proBNP for the LC2 study was measured via Alere NT-proBNP for ARCHITECT Assay (Axis-Shield Diagnostics Limited, Dundee, United Kingdom). This is a Chemiluminescence-Microparticle-Immunoassay (CMIA) in which values >125 pg/mL are considered abnormal.

For the LC1 study, NT-proBNP was measured by immunoassay (Elecsys 2010, Roche, Diagnostics GmbH, Mannheim, Germany). The mean NT–proBNP value for 100 age- and gender-matched healthy blood donors was used as a reference for the LC1 patient data (mean ± SEM = 36 ± 5 pg/mL, 99% confidence interval for upper bound = 43 pg/mL) [12].

#### 2.5.3 Echocardiography

Two-dimensional transthoracic echocardiography was performed in all patients using EPIQ 7 (Philips, Amsterdam, Netherlands). It was decided not to include echocardiography data in the present analysis because of the heterogeneous cardiac morphology in the complex ACHD group. This often limits the interpretation of cardiac function and makes it partly subjective, especially in patients with systemic right ventricle or single ventricle physiology [17].

#### 2.5.4 Statistical Analysis

Continuous variables were summarized with means and standard deviations. Categorical variables were summarized using frequencies and percentages. Receiver operating characteristic (ROC) curves were constructed for variables of interest, and areas-under-the-curve (AUC) were calculated to identify cut-off values based on the highest levels of sensitivity and specificity for predicting HF, with an AUC ≥0.70 considered to be acceptable. Logistic regression models were also constructed, with HF as the outcome and with combinations of the top performing variables of interest entered as predictors. Predicted values from the models were used in further ROC curve analyses to determine the most parsimonious combination of variables with the greatest combined AUC. The comparison of data between LC1 and LC2 for all three CHD groups (mild, moderate, and complex) were analysed by a paired *t*-test. Independent *t*-tests were used to compare data between the three CHD groups. All analyses were conducted with SPSS v27 (IBM Corp., Armonk, NY, USA), GraphPad 9.4.0 (GraphPad Software, San Diego, CA, USA), and *p*-values < 0.05 were considered statistically significant.

## 3. Results

### 3.1 Study Population

Of the initial 364 patients in LC1, a total of 249 patients (68%, 134 male and 115 female) were recruited to participate in LC2. The remaining 115/364 (32%) patients did not participate in LC2 for the following reasons: patient could not be reached or was lost to follow-up (48/364, 13%), patient declined to participate (45/364, 12%), or patient had died (22/364, 6%). Two other patients died shortly after inclusion in LC2.

The distribution of CHD severity between the LC1 and LC2 participants did not differ significantly. CHD severity in the 364 patients (58% male, 42% female) from LC1 was mild (81, 22%), moderate (199, 55%), and severe (84, 23%), while in the 249 patients (58% male, 42% female) from LC2 it was mild (52, 21%), moderate (150, 60%), and severe (47, 19%).

Patients were further classified according to their diagnosis and lesion complexity. Table 1 shows patient classification based on diagnosis, as well as the patient demographics and prevalence of HF at initial assessment (LC1-HF), and in patients who developed HF by the start of LC2 (LC2-HF). A total of 57 patients (23%) had already developed HF at LC1, while another 67 of the remaining 192 patients (35%) developed HF during the follow-up period. Of note, patients with ventricular septal defect (VSD) closure had the lowest risk of developing HF (10%), whereas all patients with single ventricle physiology (Fontan) had developed HF by LC2.

**Table 1.** **Incidence of heart failure (HF) according to the underlying heart defect at initial assessment (LC1), and the prevalence of new HF cases after 15 years of follow-up (LC2)**.

Type of heart defect	N (female)	LC1-HF (female) %	Age-LC2 (mean ± SD)	LC2-HF (female)	New HF %	*p*
Atrial septal defect	15 (9)	1 (0) {4}	36 (8)	2 (1)	14	0.30
Ventricular septal defect	21 (9)	1 (0) {3}	39 (9)	2 (1)	10	0.28
AV septal defect	12 (7)	2 (1) {13}	40 (8)	3 (2)	30	0.43
Pulmonary valve disease	14 (6)	2 (1) {11}	40 (10)	3 (2)	25	0.44
Aortic valve disease	27 (4)	8 (0) {22}	42 (9)	11 (2)	58	0.10
Coarctation of the aorta	38 (16)	13 (5) {27}	39 (7)	8 (2)	32	0.10
D-TGA	19 (5)	3 (0) {9}	36 (5)	9 (2)	56	0.001
Tetralogy of Fallot	51(22)	11 (6) {12}	45 (9)	17 (6)	43	0.01
Fontan procedure	9 (5)	4 (2) {24}	40 (8)	5 (3)	100	0.11
Miscellaneous	43 (22)	12 (6) {18}	38 (7)	7 (4)	23	0.78
Total	249 (105)	57 (21) {16}		67 (25)	35 (67 of 192)	0.0009

AV Septal defect, Atrioventricular septal defect; D-TGA, dextro-Transposition of the great arteries after surgical procedure except arterial switch; HF%, patients who developed HF from LC1 to LC2 in relation to the patients who did not have HF within this time frame; LC1-HF, number of patients who were on heart failure medication at LC1; LC2-HF, number of patients who developed HF between LC1 and LC2; SD, standard deviation. *p* indicates LC1-HF vs. LC2-HF.

Table 2 (Ref. [18]) shows the classification of patients according to lesion severity, as outlined by the 2020 ESC Guidelines for the management of ACHD [14]. Fifty-two (21%) had mild CHD, 150 (60%) had moderate CHD, and 47 (19%) had complex CHD. Patient demographics at the time of clinical work-up for LC1 and LC2 are also shown in Table 2. There was no significant difference in mean age between the three ACHD groups (*p* = 0.160).

**Table 2.** **Cardiac evaluation at LC2 according to CHD complexity [18]**.

	Mild-CHD	Moderate-CHD	Complex-CHD
	LC2 (*LC1*) {%}	LC2 (*LC1*) {%}	LC2 (*LC1*) {%}
Number of patients	52 (*81*) {64%}	150 (*199*) {75%}	47 (*84*) {56%}
Male/female (LC2)	27/25	87/63	30/17
Age (years)	39 ± 9 (*25 ± 9*)	41 ± 9 (*27 ± 9*)	38 ± 7 (*24 ± 7*)
${\mathrm{VO}}_{2\,m\,a\,x\%}$	85 ± 22 (*80 ± 17*)	86 ± 22 (*76 ± 18*)	72 ± 21 (*67 ± 13*)
NT-proBNP (pg/mL)	142 ± 114 (*80 ± 60*)	182 ± 267 (*103 ± 147*)	560 ± 748 (*300 ± 363*)
QRS (ms)	112 ± 27 (*103 ± 30*)	132 ± 34 (*121 ± 31*)	130 ± 26 (*115 ± 31*)
Patients with HF at LC1	6 of 52	35 of 150	17 of 47
Patients without HF at LC2	38	73	13
Patients with new HF at LC2 (%)	8 of 46 {17%}	42 of 115 {37%}	17 of 30 {57%}

Data are displayed as the mean ± standard deviation. Numbers in italic refer to LC1. NT-proBNP, N-terminal pro brain natriuretic peptide; Patients with HF at LC2, number of patients at LC2 who had developed HF since LC1; QRS, QRS complex duration; ${\mathrm{VO}}_{\text{2max\%}}$, percentage of predicted peak oxygen consumption at exercise testing.

### 3.2 New Presentations with HF 

As stated above, 67 of the 192 patients (35%) who did not have HF at LC1 later developed HF during the follow-up in LC2.

Table 2 shows the distribution of new HF cases according to lesion complexity. Of note, the percentage of patients with new HF increased as the lesion complexity increased (mild CHD: 17%, moderate CHD: 37%, complex CHD: 57%). Significant differences (*p *< 0.001) were found between the mild vs. moderate, mild vs. complex, and moderate vs. complex groups.

### 3.3 NT-proBNP Levels 

**Fig. 1.**
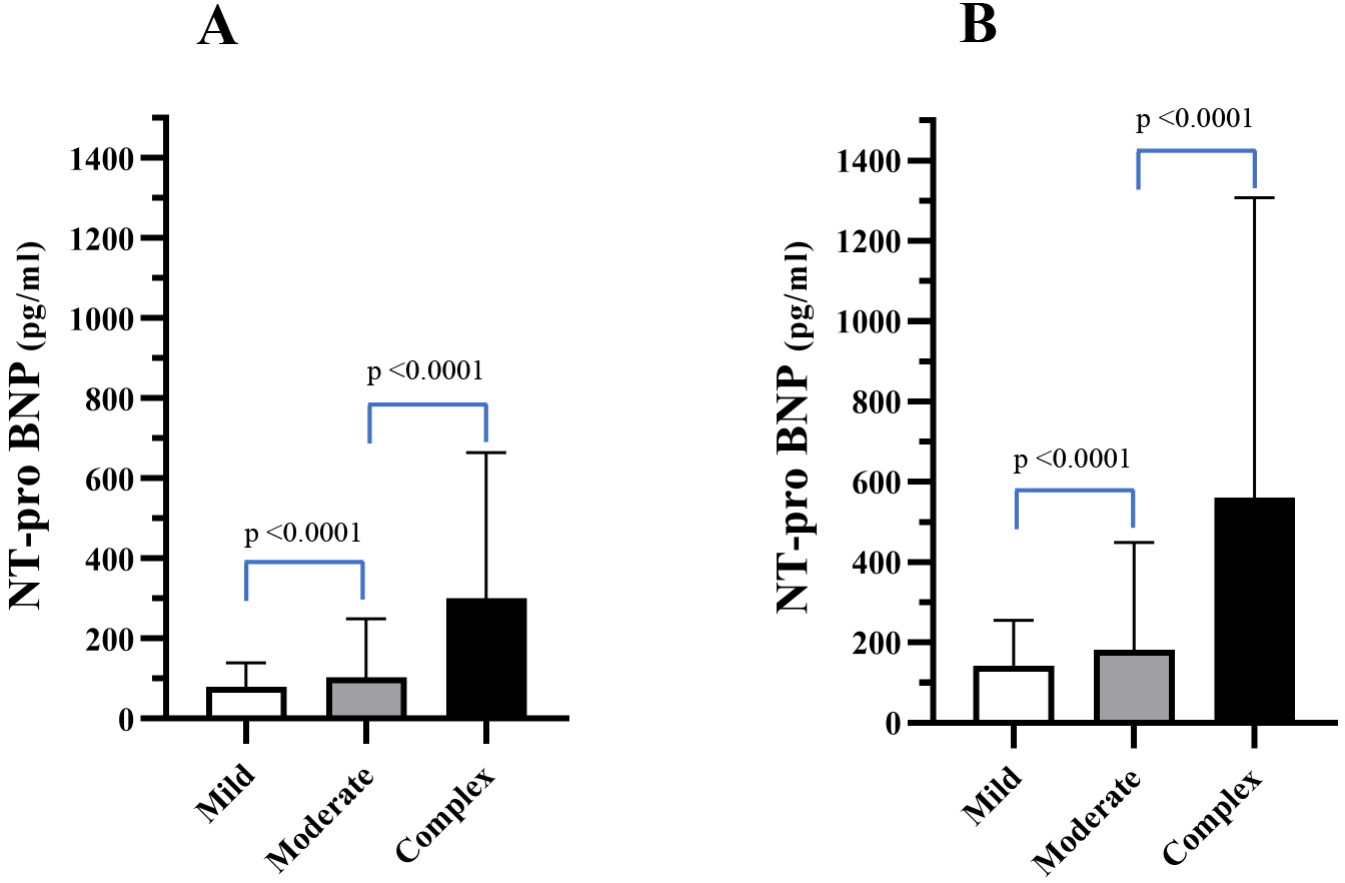
**NT-proBNP levels (pg/mL) for mild, moderate, and severe CHD at LC1 (A) and LC2 (B)**. Mean ± SD values are shown.

Table 2 and Fig. 1A,B show the levels of NT-proBNP in the three ACHD groups at LC1 and LC2, respectively. Significant increases in NT-proBNP were observed in all three groups at LC2 compared to LC1. Differences between the three groups at LC2 remained significant (mean complex ACHD = 560 pg/mL, mean moderate CHD = 182 pg/mL, and mean mild CHD = 142 pg/mL; *p *< 0.001).

Table 3 shows the NT-proBNP levels at LC1 in patients who later developed HF in LC2. These were already significantly higher compared to patients who did not develop HF.

**Table 3.** **Comparison between patients who developed HF between LC1 and LC2 and those who did not develop HF. This data was acquired at LC1 and is displayed as the mean ± SD**.

	HF patients	Non-HF patients	*p*
Number of patients	67	125	
${\mathrm{VO}}_{\text{2max}}$ (%)	74 ± 16	80 ± 19	0.03
NT-proBNP (pg/mL)	126 ± 121	88 ± 117	0.03
QRS (ms)	121 ± 34	110 ± 29	0.10

${\mathrm{VO}}_{\text{2max\%}}$, Percentage of predicted peak oxygen consumption at exercise testing; NT-proBNP, N-terminal pro brain natriuretic peptide; QRS, QRS complex duration.

### 3.4 Exercise Testing

**Fig. 2.**
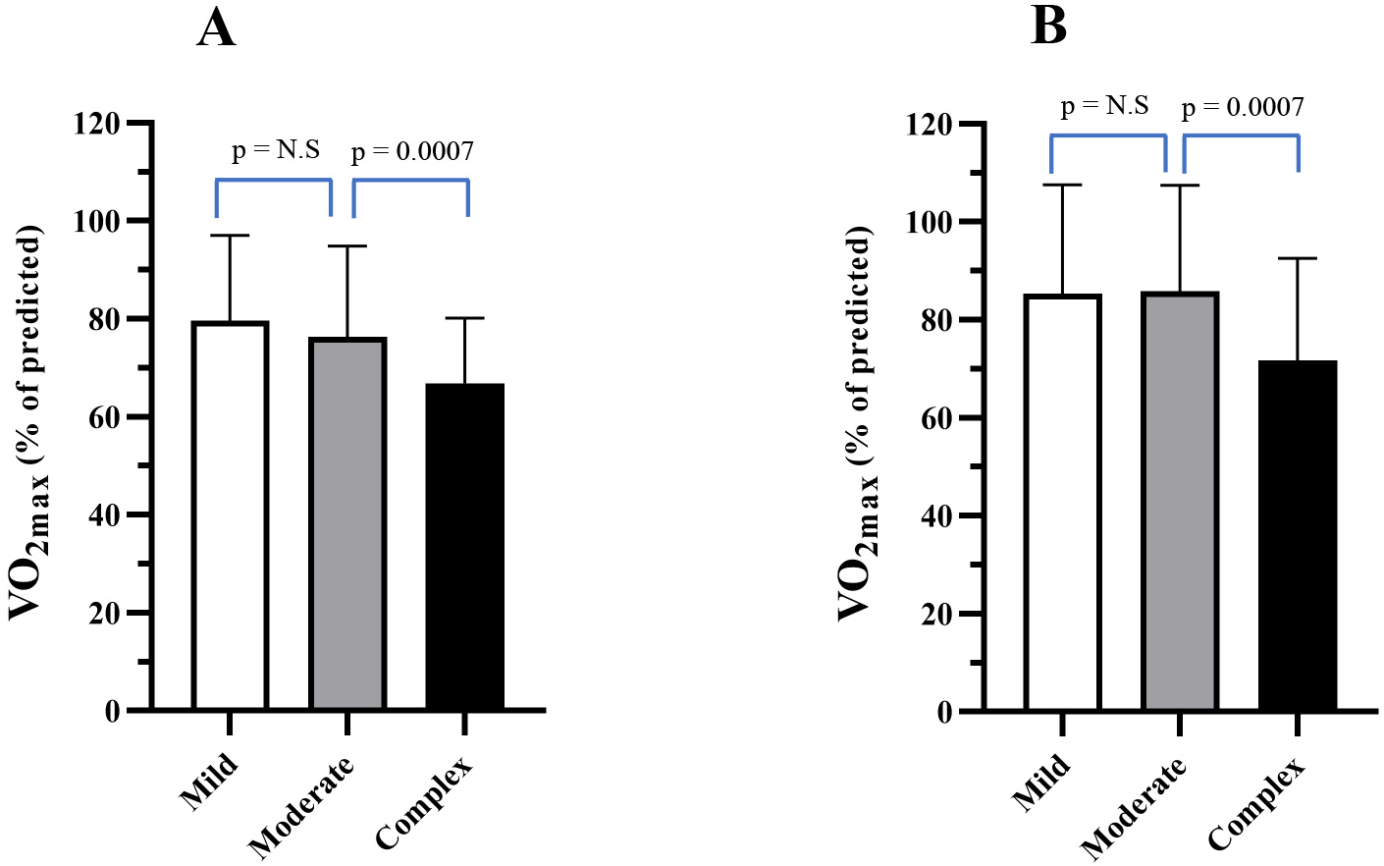
**${\mathrm{VO}}_{\text{2max\%}}$ (% predicted) for mild, moderate, and severe CHD (mean ± SD) at LC1 (A) and LC2 (B)**.

Table 2 and Fig. 2A,B show the percentage of predicted ${\mathrm{VO}}_{\text{2max}}$ in all three groups at LC1 and LC2, respectively. No significant difference in the mean ${\mathrm{VO}}_{\text{2max\%}}$ between patients with mild and moderate ACHD (85% and 86%, respectively; *p* = 0.880) was found at LC2. However, the mean ${\mathrm{VO}}_{\text{2max\%}}$ was significantly lower in complex CHD patients (72%, *p *< 0.001) compared to the other two groups at LC2. Table 3 shows that patients who developed HF by LC2 already had significantly lower ${\mathrm{VO}}_{\text{2max\%}}$ at LC1 compared to patients who did not develop HF by LC2. The mean ${\mathrm{VO}}_{\text{2max\%}}$ for all patients from LC1 was found to be significantly lower than that of all patients from LC2 (74% vs. 81% respectively; *p* = 0.048).

### 3.5 QRS Duration

**Fig. 3.**
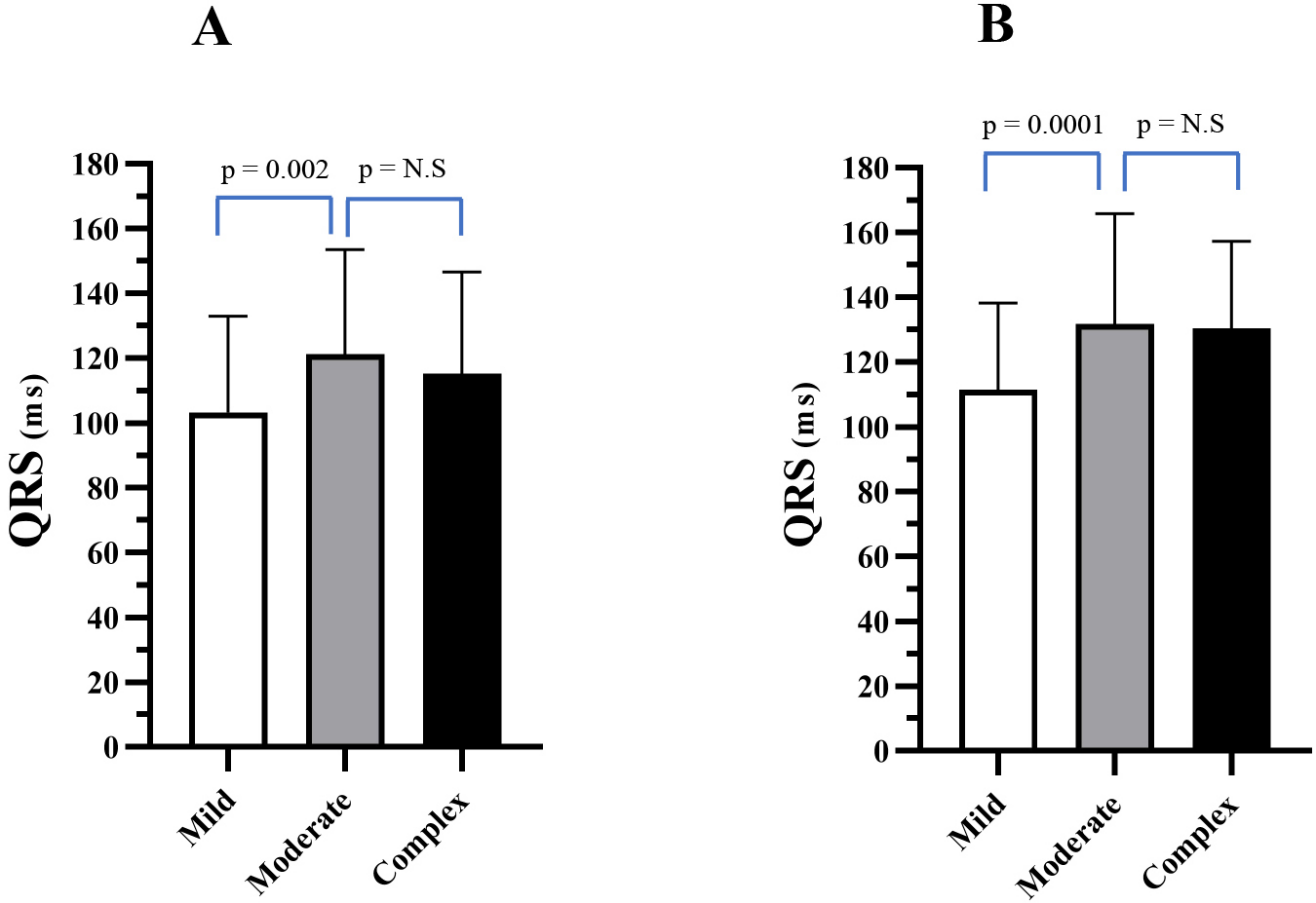
**QRS duration (ms) for mild, moderate, and severe CHD (mean ± SD) at LC1 (A) and LC2 (B)**.

Table 2 and Fig. 3A,B show the QRS duration in all three groups at LC1 and LC2, respectively. In LC1 there was no significant difference in mean QRS duration between patients with moderate and complex ACHD (121 vs. 115 ms, respectively; *p* = 0.4). However, the QRS complexes of these patients was significantly longer compared to that of mild ACHD patients (103 ms; *p* = 0.002). In LC2, the mean QRS duration in all three groups was significantly longer compared to the equivalent group from LC1: 112 ms for mild (*p *= 0.03), 130 ms for moderate (*p* = 0.0001), and 132 ms for complex CHD (*p *= 0.001). The QRS duration of LC2 patients with mild CHD was shorter than that of the other two groups (*p *= 0.001), but there was no significant difference between the moderate and complex CHD patients (*p* = 0.99).

### 3.6 Combining Parameters to Predict HF

**Fig. 4.**
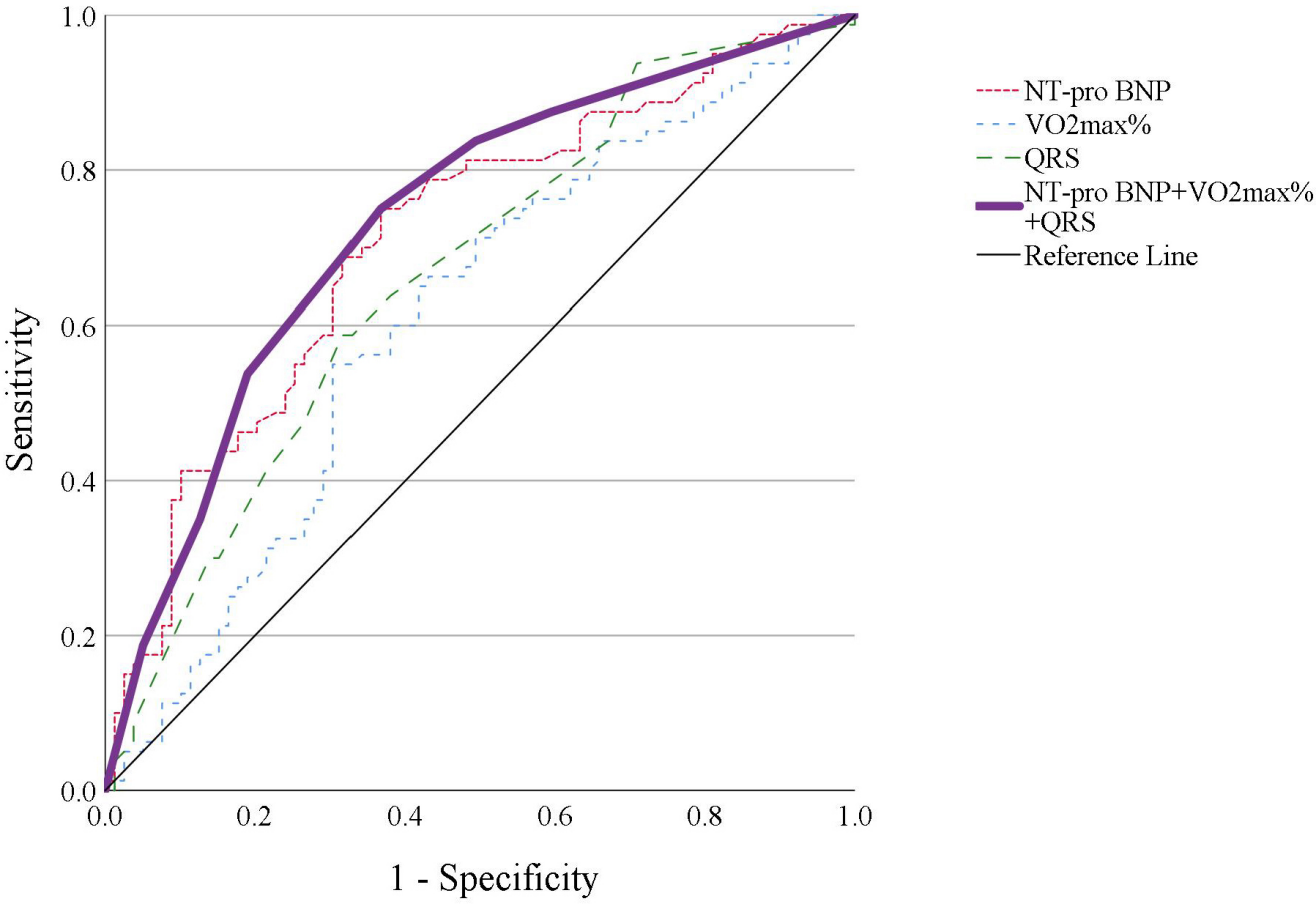
**Results of AUC, sensitivity, and specificity analyses for the development of HF in all three ACHD groups for individual parameters, and in combination**. No statistically significant differences were found between results for the combined parameters and the individual parameters of NT-proBNP (*p* = 0.630), ${\mathrm{VO}}_{\text{2max\%}}$ (*p* = 0.238) and QRS (*p* = 0.404).

The following parameters were found to be the best predictors for the development of HF between LC1 and LC2: NT-proBNP >73 pg/mL, ${\mathrm{VO}}_{\text{2max\%}}$ <73% of predicted, and QRS >120 ms. It is also important to note that NT-proBNP >73 pg/mL at LC1 was 1.7-fold higher than the 99% confidence interval (43 pg/mL) for healthy individuals. Table 4 shows the AUC, sensitivity, and specificity for the development of HF in all three ACHD groups, and separately for patient groups with mild and moderate CHD. To find the strongest predictor of HF, the AUC of each parameter was calculated individually and with all possible combinations. Although each parameter alone showed good prediction for developing HF, the combination of all three parameters resulted in the highest AUC (0.75), sensitivity (0.75), and specificity (0.63) for all lesion complexity. Although the results obtained with the combined parameters and with each individual parameter were not statistically different, the best AUC was obtained by using the combination of all three parameters, as seen in Fig. 4.

**Table 4.** **Results of AUC, sensitivity, and specificity analysis for the development of HF in the three ACHD groups combined and individually for patients with mild and moderate CHD**.

Parameter	Patient group	AUC	Sensitivity	Specificity
NT-proBNP	All Patients	0.71	0.64	0.67
	Only mild + moderate	0.66	0.63	0.63
${\mathrm{VO}}_{\text{2max\%}}$	All Patients	0.64	0.56	0.67
	Only mild + moderate	0.62	0.60	0.50
QRS	All Patients	0.65	0.56	0.69
	Only mild + moderate	0.68	0.60	0.68
NT-proBNP + ${\mathrm{VO}}_{\text{2max\%}}$	All Patients	0.71	0.63	0.67
	Only mild + moderate	0.68	0.56	0.70
NT-proBNP + QRS	All Patients	0.71	0.69	0.68
	Only mild + moderate	0.71	0.63	0.71
${\mathrm{VO}}_{\text{2max}}$ + QRS	All Patients	0.66	0.77	0.52
	Only mild + moderate	0.67	0.76	0.53
NT-proBNP + ${\mathrm{VO}}_{\text{2max\%}}$ + QRS	***All Patients***	***0.75***	***0.75***	***0.63***
	***Only mild + moderate***	***0.75***	***0.73***	***0.66***

The following parameters were included for this calculation: NT-proBNP greater than 1.7 times the upper normal limit (>73 pg/mL), ${\mathrm{VO}}_{\text{2max\%}}$ <73% of predicted, and QRS >120 ms. Italic and bold show the best AUC, sensitivity and specificity.

## 4. Discussion

The timely identification of patients who are at risk of developing HF is critical to improving their outcome through appropriate therapeutic intervention. Several international HF societies and associations recently published a consensus statement that emphasizes the importance of adding objective parameters to the clinical findings in order to establish a universal definition and classification of HF [19]. HF poses a major challenge for the management of ACHD patients, including its definition, pathophysiologic understanding, healthcare planning, and the provision of evidence-based medical therapies to improve outcomes [20]. The current study provides long-term follow-up data of previously described ACHD patients with mild, moderate, and complex CHD. We were able to identify objective and easily reproduceable parameters to predict the development of HF in this patient cohort over a 15-year interval.

### 4.1 Biomarkers

Biomarkers have major significance for the diagnosis of HF. Giannokoulas *et al*. [8] reported that elevated BNP levels (>78 pg/mL) were a predictor of death in ACHD patients, with a sensitivity of 100% and a specificity of 76.3%. None of the patients in their study with a BNP level <78 pg/mL died during an 8-year follow-up period. In a study of 595 clinically stable ACHD patients, Baggen *et al*. [21] found that NT-proBNP >14 pmol/L was strongly associated with severe cardiovascular events such as HF and death. The present data confirms that increased NT-proBNP level is a good surrogate biomarker for cardiovascular risk stratification, including for HF in ACHD patients. We found significant differences in NT-proBNP levels between the three CHD groups in LC1, with the highest levels in the complex group, followed by the moderate and mild groups. During the 15-year follow-up period, a significant number of patients with moderate and complex CHD developed HF. A two-fold increase in NT-proBNP levels between LC1 and LC2 was observed in all three CHD groups. The increased NT-proBNP levels are likely attributable to HF rather than aging, since approximately 35% of patients developed HF between LC1 and LC2 [22, 23].

### 4.2 Exercise Testing

${\mathrm{VO}}_{\text{2max}}$ is considered to be a reliable parameter for the measurement of cardiorespiratory capacity [24]. In a study of 1375 ACHD patients over a period of 10 years, Inuzuka *et al*. [9] demonstrated that a combination of peak oxygen uptake and heart rate reserve was related to midterm survival. Based on a random survival forest analysis, the authors found that 64% of predicted ${\mathrm{VO}}_{\text{2max}}$ was the optimal cut-off value for the prediction of 5-year survival. Furthermore, Diller *et al*. [25] identified that ${\mathrm{VO}}_{\text{2max}}$ was a predictor for the hospitalization and death of ACHD patients. Our study identified a threshold of <73% of predicted ${\mathrm{VO}}_{\text{2max}}$ as the optimal cut-off for predicting the development of HF.

Patients in LC1 with complex ACHD already had significantly reduced ${\mathrm{VO}}_{\text{2max\%}}$ compared to other patients, with this difference remaining unchanged after 15 years of follow-up. Interestingly, the mean ${\mathrm{VO}}_{\text{2max\%}}$ of all three patient groups was higher in LC2 than in LC1, probably because of the different exercise protocols used. In support of this, Michalik *et al*. [26] reported that the RAMP protocol resulted in higher ${\mathrm{VO}}_{\text{2max}}$ values during shorter duration of testing compared to the STEP protocol.

### 4.3 QRS Complex Duration

Widening of the QRS complex has been identified as an independent predictor of adverse outcomes in ACHD [10, 27]. Müller *et al*. [28] conducted a multicenter retrospective investigation on 875 patients with tetralogy of Fallot. These authors reported that patients with a QRS duration of ≥170 ms and a predicted ${\mathrm{VO}}_{\text{2max}}$ ≤65% had an 11.4-fold increased risk of death or sustained ventricular tachycardia. In the present study, patients with moderate or complex CHD at LC2 had a significantly prolonged QRS duration (132 ms and 130 ms, respectively), whereas the QRS complex remained within normal limits in mild ACHD patients (112 ms). In most cases, QRS prolongation in patients from our cohort was due to complete right bundle branch block (RBBB), which has previously been associated with myocardial dysfunction and the development of HF [29, 30].

In adults without CHD, QRS prolongation ≥120 ms is present in 14% to 47% of patients with HF. Left bundle branch block is far more common than RBBB in these patients, in contrast to our CHD population. It is well established that left-sided intraventricular conduction delay is associated with more advanced myocardial disease, worse left ventricular (LV) function, poorer prognosis, and higher all-cause mortality compared with narrow QRS complex [31].

The progression of QRS complex duration in our patients (mainly in the moderate and complex CHD groups) from LC1 to LC2 is assumed to reflect decreasing cardiac function as a pattern of electro-mechanical interaction. In patients without CHD, progressive increases in QRS duration were shown to predispose HF patients to an increased risk of ventricular tachyarrhythmias [32, 33]*.*

In order to identify surrogate parameters for predicting the development of HF, we applied logistic regression models to combinations of the top-performing variables of interest. Regardless of the severity of the underlying HF, the best result was found to be a combination of three parameters: NT-proBNP, ${\mathrm{VO}}_{\text{2max\%}}$, and QRS complex duration (AUC = 0.75, sensitivity = 75%, and specificity = 63%). For example, patients with NT-proBNP >1.7 times the upper limit of normal (ULN) and otherwise normal values for ${\mathrm{VO}}_{\text{2max}}$ and QRS complex duration had a 71% probability of developing HF between LC1 and LC2. However, the risk of developing HF increased to 75% if the patients fulfilled all three criteria of NT-proBNP >1.7 ULN, ${\mathrm{VO}}_{\text{2max\%}}$ <73% of predicted, and QRS >120 ms.

Our findings indicate that assessment of these parameters in ACHD patients could provide predictive information on patients who are at high risk of developing HF. These parameters have the advantage of being investigator-independent and of not requiring a deep knowledge of CHD. Hence, they might be a useful screening tool to indicate the need for referral to large ACHD centers.

## 5. Conclusions

To the best of our knowledge, this study is unique as it analyzes a large cohort of ACHD patients with a wide variety of CHD over a 15-year period. This allowed us to identify robust parameters for predicting the development of HF. It is important to note that these parameters can be applied for risk stratification of all ACHD patients, regardless of the type and complexity of their underlying CHD. Presently, HF is often not identified promptly in patients with ACHD. The increasing number of hospitalizations of ACHD patients, particularly due to HF, is a growing burden on the healthcare system [34, 35]. The present study found that investigator-independent parameters consisting of a laboratory test, exercise test and ECG can be used to construct prediction models that help to identify ACHD patients who are at high risk of developing HF. These patients may benefit from early referral and close follow-up by ACHD specialists, thereby allowing sophisticated monitoring and timely interventions.

## 6. Study Limitations

Although the number of patients from the LC1 study who were lost to follow-up was quite low (13%), 32% of the original cohort did not participate in the current study. This may have affected the results, since the prevalence of HF in patients with ACHD is unknown [13]. Notably, more patients with mild CHD were lost to follow-up than patients with moderate or complex CHD. Multiple factors could have contributed to this, including the patients’ belief that further cardiological follow-up was not required, refusal to accept CHD as a life-long issue, moving to an area without a known ACHD specialist, or simply changing the place of residence. Another limitation was the different immunoassays used during the LC1 and LC2 studies. The NT-proBNP immunoassays used in LC1 were different to those used in LC2. However, the value of >73 pg/mL used as the threshold to define a predictor of HF was 1.7-fold higher than the 99% confidence interval (43 pg/mL) for the level in healthy individuals. Lastly, metabolic stress tests were performed using a bike ergometer, but the use of different settings (ramp vs. conventional protocol) and type of equipment may have affected the calculation of percent of predicted ${\mathrm{VO}}_{\text{2max}}$. These factors may explain why the ${\mathrm{VO}}_{\text{2max\%}}$ values in all three patient groups were higher in LC2 compared to LC1.

## Data Availability

The datasets used and/or analyzed during the current study are available from the corresponding author on reasonable request.

## Abbreviations

ACHD, Adult Congenital Heart Defect; CHD, Congenital Heart Defect; HF, Heart Failure; LC1, Life chances 1: Assessment of patients between 2003 and 2004; LC2, Life chances 2: Assessment of patients between 2018 and 2019; NT-proBNP, N-terminal pro Brain Natriuretic Peptide; ${\mathrm{VO}}_{\text{2max}}$, Peak Oxygen Consumption; ${\mathrm{VO}}_{\text{2max\%}}$, Percentage of predicted peak Oxygen Consumption.
